# Protocol for GET FIT Prostate: a randomized, controlled trial of group exercise training for fall prevention and functional improvements during and after treatment for prostate cancer

**DOI:** 10.1186/s13063-021-05687-7

**Published:** 2021-11-06

**Authors:** Kerri M. Winters-Stone, Fuzhong Li, Fay Horak, Nathan Dieckmann, Arthur Hung, Christopher Amling, Tomasz M. Beer

**Affiliations:** 1 Knight Cancer Institute, School of Medicine, Oregon Health & Science University, 3181 SW Sam Jackson Park Road, Portland, OR 97239 USA; 2 School of Nursing, Oregon Health & Science University, Portland, OR USA; 3 Oregon Research Institute, Eugene, OR USA; 4 Department of Neurology, School of Medicine, Oregon Health & Science University, Portland, OR USA; 5 Department of Psychiatry, School of Medicine, Oregon Health & Science University, Portland, OR USA

**Keywords:** Prostate cancer, Falls, Frailty, Exercise, Physical activity

## Abstract

**Background:**

Many prostate cancer survivors are treated with androgen deprivation therapy (ADT), but these therapies may increase frailty, worsen physical functioning, and increase fall risk. While exercise may counter functional declines associated with ADT, no studies have tested whether and which type of exercise may reduce falls and frailty. The purpose of this trial is to compare the relative efficacy of strength training versus tai ji quan training against each other and to a stretching control group on falls, frailty, and physical functioning in men expose to ADT for prostate cancer.

**Methods:**

Prostate cancer survivors treated with ADT (*N* = 360) who have fallen in the past year or are at risk of a fall based on validated risk factors will be recruited to participate in this single-blind, parallel group, randomized trial. Participants will be randomized to one of three supervised, group training programs: (i) strength training, (ii) tai ji quan training, or (iii) stretching (control), that train 3×/week for 6 months. Outcomes are assessed at baseline, 3 (mid-intervention), 6 (immediately post-intervention), and 12 (follow-up) months. The primary outcome is falls assessed by monthly self-report. Secondary outcomes include the following: frailty (low lean body mass (by bioelectrical impedance analysis), exhaustion (by SF-36 vitality scale), low activity (by CHAMPS physical activity survey), slowness (by 4 m usual walk speed), and weakness (by chair stand time)); objective and subjective measures of physical function will also be collected. Negative binomial regression models will be used to assess differences in falls between groups, while mixed effects modeling will be used to compare the relative efficacy of training group on secondary outcomes.

**Discussion:**

Exercise represents a non-pharmacologic approach to mitigate the problem of falls experienced among men treated with ADT. By engaging in appropriate exercise, men may be able to avoid or delay falls, frailty, and disability associated with their cancer treatment. Findings of the trial are expected to inform clinical practice about how exercise could be prescribed as part of cancer care for prostate cancer survivors prescribed ADT.

**Trial registration:**

ClinicalTrials.gov NCT03741335. Registered on November 18, 2018.

**Supplementary Information:**

The online version contains supplementary material available at 10.1186/s13063-021-05687-7.

## Background

**Fig. 1:**
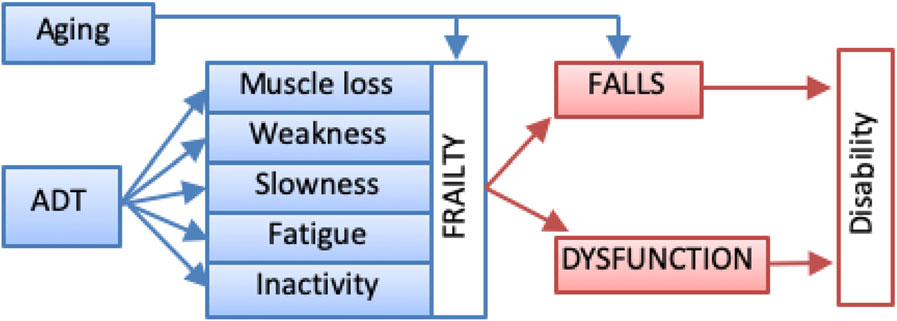
Accelerated aging from ADT and the consequences

Nearly one million prostate cancer survivors in the USA receive androgen deprivation therapy (ADT) to reduce tumor androgen exposure [1–3]. Although ADT is beneficial for cancer survival, side effects from ADT may lead to serious health consequences including falls, frailty, and dysfunction that contribute to morbidity and mortality [4–9]. ADT is associated with an increased risk of falls and frailty, even after treatment stops [8]. Recent evidence indicates that prostate cancer survivors exposed to ADT are significantly more likely to report a history of falls, injurious falls, frailty, and dysfunction compared to prostate cancer survivors never on ADT [4–9]. ADT is associated with muscle loss, weakness, fatigue, slowness, and inactivity [10–12] which are linked to falls in adults without cancer, and constitutes the same elements of the frailty phenotype that predicts poor health outcomes [13] (Fig. 1). Findings show that the rate of falls, including those resulting in injury, are 2–3 times higher among prostate cancer survivors who receive ADT compared to men who never receive this treatment or to otherwise healthy, older men. In addition, prostate cancer survivors, whether treated currently or in the past with ADT, have a 5 to 6 times higher risk of recurrent falls and 9 to 10 times higher risk of frailty than men never on ADT [8]. Nearly half of prostate cancer survivors are treated with ADT [3], placing millions of men worldwide at increased risk for these side effects.

Currently, clinical practice has no effective solution to the rising problems of falls and frailty from ADT. Despite the high risk associated with ADT, no fall or frailty prevention strategies, especially exercise-based modalities, have been developed in this clinical population and clinical guidelines are bereft of recommendations to manage these life-threatening consequences of treatment. Exercise has been shown to offer symptomatic relief from side effects of cancer treatment and improve quality of life among cancer survivors, but the potential benefits of exercise to prevent falls and frailty associated with ADT are unknown [14]. Based on research in older adults without cancer, either tai ji quan (also known as “tai chi”) or strength training exercise could prevent falls and frailty in prostate cancer survivors on ADT. Tai ji quan and strength training each reduce falls in older adults without cancer by targeting different mechanisms: balance control or muscle strength, respectively [15]. Since the precise reasons that ADT increases falls are not yet known, a rigorous head-to-head comparison of two traditional types of exercise that are grounded in the evidence from older adults without cancer must also be evaluated as a fall prevention strategy for prostate cancer survivors.

The primary objective of this study is to determine and compare the efficacy of tai ji quan training and strength training in reducing the incidence of falls in prostate cancer survivors on ADT, as compared to a stretching control group. Our secondary objectives are to compare the efficacy of tai ji quan training and strength training to reduce frailty and dysfunction in prostate cancer survivors on ADT, to determine how well training effects persist over a 6-month period, and to identify patterns that predict the type of men who benefit most from exercise training. We hypothesize that tai ji quan and strength training groups will each reduce the incidence of falls, reduce the prevalence of frailty, and improve physical function, as compared to a control group. The relative efficacy of each type of training on these outcomes is not currently known, but will be tested in this study.

## Methods

### Study design and setting

The GET FIT Prostate trial (*g*roup *e*xercise *t*raining for *f*all prevention and functional *i*mprovements during and after *t*reatment for *prostate* cancer) is a 3-arm, single-blind, parallel group, randomized controlled trial comparing tai ji quan and strength training against each other and vs. a stretching placebo control. All three study arms will train concurrently in supervised group exercise classes for 6 months. Data collection will occur at baseline, 3 months (midpoint of exercise), 6 months (end of supervised training), and at 1 year (6 months post-supervised training). Interventions and outcomes assessments will occur at Oregon Health & Science University in Portland, Oregon, and at community sites in Salem (Salem Hospital), Bend (St. Charles Medical Center), and Eugene (Oregon Research Institute), Oregon. These planned research activities can also occur at local community sites which will allow the trial to be conducted in multiple regions across the state, to meet enrollment goals and enhance recruitment of a broader and more diverse group of participants. The study is approved by the OHSU IRB (#18354) and registered with ClinicalTrials.gov (NCT03741335). Any modification of this protocol must be documented in the form of a protocol revision or amendment signed by the principal investigator and approved by the OHSU Knight Cancer Institute and the IRB before the revision or amendment may be implemented. The only circumstance in which the amendment may be initiated without regulatory approval is for a change necessary to eliminate an apparent and immediate hazard to the patient. In that event, the investigator must notify the IRB in writing within 5 working days after the implementation.

### Sample

Participants will be prostate cancer survivors who currently are on or have been on ADT. To be eligible men must meet the following criteria: (1) histologically confirmed prostate cancer, (2) received at least 6 months of ADT within the past 10 years, (3) report > 1 fall in the past year OR have a score on one of two physical performance tests that is associated with increased fall risk (i.e., ≥ 12.0 s to complete the 3 m timed up and go (TUG) [16], or ≥ 10.0 s to complete 5 chair stands) [17], (4) completed any other treatment (e.g., surgery, radiation, chemotherapy) at least 6 weeks prior to enrollment and not be on any concurrent prostate cancer therapy besides ADT, (5) not currently participating in moderate-vigorous-intensity lower body strength training or tai ji quan training ≥ 2 times/week for ≥ 30 min per session, (6) no cognitive difficulties that limit ability to answer survey questions or participate in exercise classes and performance tests, (7) no medical condition, disorder, or take medication that contraindicates participation in moderate intensity exercise, (8) are able to communicate in English, and (9) willing to attend > 75% of the intervention classes at the designated time of day, days of the week, and/or location for exercise training. All men must also receive medical clearance for participation in moderate intensity exercise.

### Power and sample size

Sample size is based on the ability to detect a 47% difference in the number of falls between each experimental exercise arm and the control group using our estimates derived from clinical trials of tai ji quan in older adults using a similar study design [18, 19]. The effect size for tai ji quan is used to estimate power for both interventions because we have these data available from our prior work and effects sizes for strength training are similar to tai ji quan [20]. These estimates will also allow us to determine if strength training is as effective as tai ji quan at reducing falls. Using PASS 2008 [21], based on a negative binomial regression model [22], at alpha = .05, the variance shared among the dummy vectors representing treatment (*R*^2^ = .25), and an estimate of the overdispersion parameter (Phi) of 2.5, a sample size of *n* = 300 (*n* = 100 per group) participants will provide 80% power to detect at least a 47% reduction in the fall incidence rate over 6 months by being in either of the 2 exercise groups versus control. To protect against an estimated attrition of ~ 17% during the intervention period, 120 participants will be randomized per group (total sample size: *N* = 360). The attrition estimate is conservatively based on the highest attrition rate from our prior strength training trials in prostate cancer survivors [23, 24]. Though the total number of falls is the primary endpoint, the sample size of *N* = 300 also provides sufficient power to detect differences between exercise intervention and control groups on secondary outcomes of frailty and physical function. With a sample of *n* = 100 per group, we have a sufficient sample to detect a difference in 2.5% of lean body mass [25, 26], a 4-s faster chair stand time [24, 26], a 1.1-s faster gait speed [26], and a 9% difference in self-report physical function scores [24].

### Recruitment

We plan a 40-month enrollment period to recruit 360 men into the trial, which is a similar enrollment rate to our previous studies in prostate cancer survivors on ADT [27]. Men will be enrolled into 8 waves of 45 men to maintain reasonable class sizes (~ 15 men per class). Our primary recruitment strategy will be through the Oregon State Cancer Registry (OSCaR), which we have used for previous studies to recruit 50–80% of participants. At the time of funding, records indicated that there were 6112 living prostate cancer survivors who meet the study age criteria in the targeted recruitment areas. Based on estimates of ADT use, we expect 45% of this pool to be current or past ADT users. Of that group, we estimate ~ 25% may not meet eligibility criteria for fall history or risk, cognitive limitations, or medical contraindications to exercise. Of the remaining 2062 men, we expect to enroll up to 412, knowing we have enrolled ~ 20% of eligible survivors in prior work [24]. In addition to registry letters, we will recruit by clinician referral through our hospital cancer registry and community oncology clinics. Direct community recruitment will also occur via print ads, radio, social media, and presentations at cancer organizations and conferences. Finally, we will work with other area health systems and providers to obtain referrals and basic demographic data of potential participants so they may be contacted by letter or phone.

### Retention

To improve motivation to exercise and promote compliance to the study interventions, an incentive will be provided to participants at the midpoint and end of the intervention period. Participants will be provided with a potential to earn $50 at each 3-month time point ($100 for 6-month intervention) depending on their attendance at weekly exercise sessions. The amount received will depend on the average number of exercise class sessions attended per week. We will use the following compensation structure based on average attendance over each 3-month period: > 2 classes per week = $50; > 1 class per week = $40; > 0.5 classes per week = $30, < 0.5 classes per week = no compensation. In addition, there will be a $10 remuneration per testing visit to offset transportation costs.

### Procedures

**Fig. 2:**
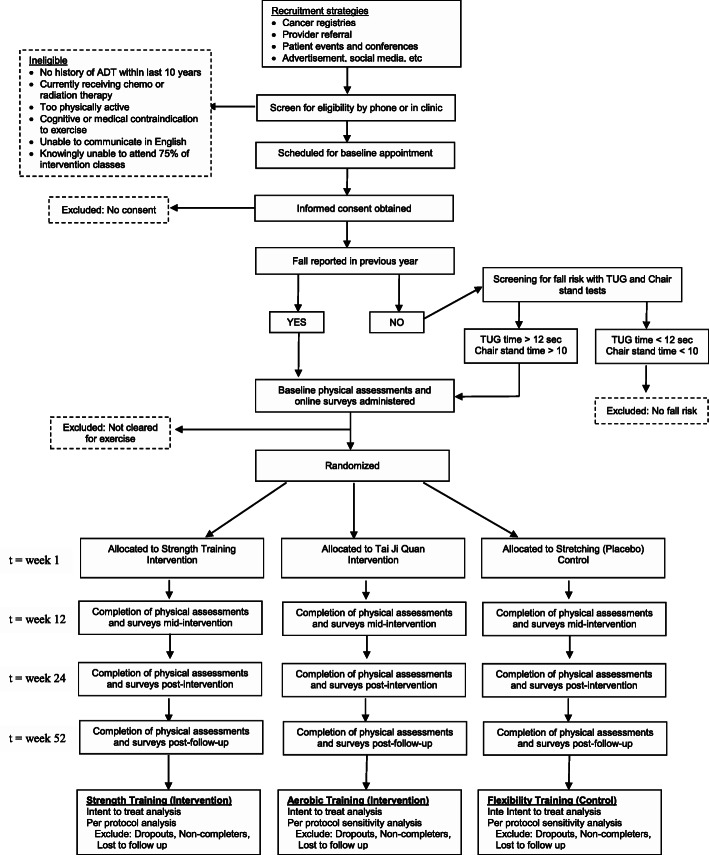
Planned participant flow through the trial

The planned flow of participants in the study is outlined in Fig. 2. Participants who express interest in the study will be screened for eligibility either by phone or in person by study staff. After initial screening, potentially eligible men will be scheduled to go to OHSU for consent, further screening, and baseline testing if eligible. Baseline screening includes review of fall history in the past year and screening performance tests for men reporting no falls (see “Sample” section for inclusion criteria). Men who meet criteria for fall history or fall risk will proceed to baseline testing where they are first enrolled by study staff and then complete body composition assessment, physical performance testing, and initial surveys. Men will complete written surveys on a computer at baseline and online for follow-up visits, unless they prefer to complete paper surveys. Staff will review surveys for completeness and follow-up with participants in person or by phone on missing data. Except for screening measures, these same measurements are repeated at 3, 6, and 12-month follow-up visits. All study outcome assessors are blinded to group assignment, which occurs after baseline testing. If a participant drops out of the research his data will be retained for analysis, but no more data will be collected past the point of withdrawal. The investigator may choose to withdraw a participant without their consent if his health changes and the study is no longer in their best interest, if new information becomes available, if he does not follow the study rules, and/or if the study is stopped by the IRB.

### Randomization and blinding

Participants will be randomly assigned to 1 of 3 groups in a 1:1:1 allocation ratio: (1) tai ji quan, (2) strength training, or (3) stretching control. To avoid confounding that could occur due to differential exercise tolerance based on ADT history, block randomization, stratifying for timing of the last exposure to ADT (> 1 year ago, ≤ 1 year ago), will be used. The biostatistician (ND) uses a computer-generated (MS Excel) random numbers table to allocate participant ID numbers to study arms in blocks of 6–9 men per study wave to ensure even assignment across waves. Allocation concealment will be ensured by placing individual assignments into sealed envelopes prior to enrollment. The allocation sequence assigns men in the order that they are scheduled for baseline testing. After completion of baseline testing, the project director gives men the sealed envelope that contains their randomly assigned group.

### Study interventions

Participants in each study group will attend 1-h classes, 3 days/week for 6 months that are led by certified exercise instructors. Class size will be limited to 15–16 participants per class so that enough individual attention is given to participants to ensure proper form and safety. Interventions will be performed at a low-moderate intensity, progressing from low to moderate intensity over the first 4–6 months, while remaining at a constant overload over the last 2 months of training. To ensure quality control over intervention delivery within and across instructors, every trainer will complete 2-day training workshop that covers how to instruct each exercise protocol, training progression, safety considerations, and research conduct specific to the exercise program. Additionally, each trainer will follow a written training protocol and will be regularly observed by study staff who monitor participant retention, exercise compliance, and instruction fidelity.

#### Strength training

Recommendations for improving muscle strength in older adults support the use of multiple-joint exercises for 1–3 sets per exercise at a weight that can be lifted 8–12 times [28]. This study strength training intervention will use weighted vests to apply resistance during lower body exercise***.*** The protocol is a progressive strength training program (Table 1) based upon our prior programs that improved neuromuscular function (strength, gait, and balance) and reduced fall risk factors in our prior studies in women with [29, 30] or without cancer [31, 32] and in our previous trials in prostate cancer survivors on ADT [23, 24, 27]. Participants will wear a weighted vest while performing exercises using functional movement patterns that challenge balance, by using muscle groups and movement involved in everyday activities (e.g., chair rises, 90° squats, side-to-side squats, toe raises, multi-direcitonal lunges, step-ups). The vest has multiple small pockets that each hold ½ pound weights, so that the intensity of the exercise can be adjusted slowly. Pockets are distributed evenly around the torso in order to add resistance in an ergonomically efficient and safe way. Weighted vests allow participants to perform functional exercises without safety risks related to balance disruption that can occur with handheld barbells and dumbbells.

**Table 1.** Planned progression of strength training over 6 months (*BW* body weight)

Month	Exercise volume		
	Intensity	Sets	Repetitions
1	4–6% BW	1–3	10–15
2	7–9% BW	1–3	10–12
3	10–13% BW	1–3	10–12
4	13–15% BW	1–3	10–12
5	15% BW	1–3	8–10
6	15% BW	2–3	8–10

##### Tai ji quan

The goal of this exercise program is to assist patients in retaining postural control and stability, with exercises specifically designed to challenge limits of stability, induce sensory integration, and train gait patterns. An integrated exercise routine consisting of 8 purposeful movement forms and a set of therapeutic movements such as displacement of body’s center of mass over the weight-bearing leg, and step initiation, locomotion, and termination. The only resistance applied during tai ji quan will be the participant’s own body weight. This program was shown efficacious [19, 33] and disseminable in community settings [34, 35]. The early stage of the program (i.e., the first 12 weeks) emphasizes primarily learning, practicing, and repeating single forms. The later stage focuses on performing individual forms in a sequence to improve postural balance and movement locomotion. Class instruction comprises learning new movements and reviewing movements learned in previous sessions.

##### Stretching control

Participants in this group will perform a series of whole body stretching exercises, according to the American College of Sports Medicine (ACSM) guidelines for flexibility training [28] with a goal for improving whole body range of motion. Stretching exercises will be performed in a seated or lying position that minimizes weight-bearing forces that might increase fitness or mobility. Many exercise trials in older adults, including ours, have used a stretching control condition and have shown no effect of this training on muscle strength [36]. Since the ACSM recommends “avoidance of inactivity” among cancer survivors to optimize quality of life [14], the increases in range of motion and sense of well-being from stretching may be viewed as a benefit for participants in this group. Men in this group will be asked to refrain from initiating new strength training or tai ji quan programs during their time in the study. Our prior exercise trial in prostate cancer survivors on ADT had a similar stretching control group and retention and compliance rates were similar to the strength training group [27]. In contrast, in our trial of partnered exercise in prostate cancer survivors and spouses, couples assigned to a usual care control group had greater attrition (25%) compared to the exercise group (0%) [23]; thus, the use of a placebo control group is an important feature of this study’s retention plan.

#### Six-month follow-up period

To evaluate the persistent effects of tai ji quan and strength training on falls and frailty, men will be followed for an additional 6 months after the 6-month supervised intervention stops. Men will continue to track their falls during the follow-up period using the same monthly reports as used during the 6-month intervention. A quarterly falls questionnaire will also be administered (3 months, 6 months, 9 months, and 12 months). We recognize that men may wish to maintain their exercise habits after formal training stops and we will not discourage them from doing so. During the follow-up period, exercise questionnaires will be used to track participation in home or community exercise programs and will be collected at the 9- and 12-month time periods. We will consider participation in community and/or home-based exercise in analysis. Finally, measures of frailty and function will be repeated at 12 months to better assess both the residual effects of the intervention programs among men who do not exercise in the follow-up period, and the influence of continued exercise on outcomes after formal training stops.

#### Participant safety during exercise

Any form of exercise carries a slight risk of injury. We will take steps to reduce the risk of injury and other issues that might limit compliance, including: (1) require physician clearance for every enrolled participant and (2) monitoring and early care of musculoskeletal symptoms which may include slight adjustments in the training program (modifying intensity or select exercises) with a goal to maintain the overall training stimulus.

### Measures

#### Primary endpoint

The focus of this study is capturing differences between exercise interventions in the change in number of falls from baseline up to 12 months. In this study, a fall is defined as unintentionally coming to rest on the ground or at some other lower level, not as a result of a major intrinsic event (e.g., stroke or syncope) or overwhelming hazard [37]. Falls over 1 year prior to baseline will be ascertained to characterize the sample and check for equality of randomization. Prospective assessment of falls will be done by collecting monthly reports [38] that are captured electronically using REDCap, which is an efficient (1–2 min completion time) and effective method used by our team and others [19, 39–41]. In two prior studies, we obtained 98% of monthly fall reports over 6 months [39, 42]. Participants will also be provided with a paper monthly falls calendar to record falls when they occur, in order to reduce issues of recall and false negatives. Each participant that records a fall will receive a phone call from the research team to confirm that each fall meets the standard definition. This will be done in order to reduce the risk of false positives and to obtain information about the fall (e.g., how it occurred) and any resultant injury. An “injurious” fall is one that results in fractures, head injuries, sprains, bruises, scrapes, or serious joint injuries, or where the participant seeks medical care [37]. The number of injurious falls, and medical care resulting from a fall, will be used to describe the study population.

#### Secondary endpoints

##### Frailty

In this study, we will assess frailty using measures that will most accurately capture frailty criteria in the prostate cancer survivor population and where cutoff scores can be appropriately determined. Frailty is defined by the Components of Frailty Phenotype, with 5 criteria (shrinking, exhaustion, low activity, slowness, and weakness) originally defined in the Cardiovascular Health Study [13]. The phenotype can now be measured more accurately with objective measures, such as lean body mass rather than self-report weight loss [43], as will be done here. A total frailty score will be calculated in the same manner as that of Fried [13]: > 3 criteria = frail; 1–2 criteria = pre-frail; 0 criteria = robust. Since each variable is a continuous measure, we will examine changes in each individual frailty component to determine how each intervention may shift frailty among our sample. Each of the 5 criteria that comprise the frailty score is described below.

##### Shrinking

Lean body mass will be measured by whole body DXA scan (Hologic-QDR Discovery Wi; APEX software, v.4.02) to operationalize shrinking. Since weight gain is a common side effect of ADT, capturing the loss of lean mass would be difficult by tracking weight loss alone. We will also measure lean mass by bioelectric impedance analysis (BIA; Imp SFB7, ImpediMed, Inc., Australia). Although DXA is the gold standard approach for measuring body composition, it is expensive and relies on technician training and experience [44]. BIA has been recommended as a technique to measure sarcopenia in the elderly [45] and is as accurate and reliable [46, 47]. If there is equal classification and sensitivity to change with BIA in this trial, this measurement technique could be used instead of DXA in future implementation trials. The cutoff score for moderate sarcopenia in men is < 10.75 kg/m^2^ based measures from men aged > 60 years in the NHANES II database [48].

##### Exhaustion

The self-reported score on SF-36 Vitality Scale will be used to characterize exhaustion for assessing frailty in cancer survivors [49]. The vitality scale has established reliability [50], has strong validity evidence in cancer survivors as a fatigue measure [51], and has shown sensitivity to change in response to exercise in adult, older adult, and clinical populations, including cancer [52–54]. We will use cutpoints of scores of less than 50.00 (normed) for prostate cancer survivors aged 50–64 years, or scores less than 40.00 (normed) for prostate cancer survivors aged > 65 years [50]. Although not part of the frailty score calculation, the Functional Assessment of Chronic Illness Therapy (FACIT) Fatigue scale will also be administered as a second fatigue measure specific to people with cancer. The questionnaire includes 13 items rated from “not at all” to “very much” over the past 7 days. Possible scores range from 0 to 52. Low values indicate no fatigue, while high values represent high fatigue [55, 56].

##### Low activity

Low activity will be measured by physical activity-related energy expenditure, or metabolic equivalents (METS), calculated from self-reported physical activity. We will use Fried’s original cutpoints for “low activity” of < 383 kcals per week spent in moderate-vigorous intensity activity measured by the 41-item Community Healthy Activities Model Program for Seniors (CHAMPS) physical activity questionnaire [57]. CHAMPS is a frequently used, highly reliable [58] measure of physical activity in older adults, including studies in cancer survivors performed by our team [29, 42, 59, 60].

##### Slowness

The study measure of slowness (i.e., walking speed) will be measured as the fastest time of two 15’ walks performed at a usual pace. Walks will be performed on an electronic gait mat to ensure accurate timing. We will use Fried’s original cutpoints for “slowness” in older men of times > 7 s for height > 173 cm, or times > 6 s for height < 173 cm.

##### Weakness

In contrast to grip strength used by Fried in the original phenotype [13], lower body strength will be used in this study. Lower body strength is more closely linked to falls, mobility, and dysfunction and can be easily obtained using the well-established timed chair stand test, measured as seconds required to rise from chair 5 times [61, 62]. A chair stand time > 12 s has been shown to predict a 2.4 increased risk of falls in older adults, which will be the cutoff applied in this study for “weakness” [17]. Chair stand time will be used as a measure of intervention fidelity in the strength training group.

Another secondary outcome in this study is physical function, which will be measured here both objectively (mobility, balance) and subjectively (perceived function by self-report).

##### Functional mobility

The timed up and go (TUG) test is a reliable [63] and widely accepted clinical measure of mobility that evaluates the time that it takes a person to rise from a chair, walk 3 m, turn around a cone, and return and sit in the chair [64]. Slower TUG times are associated with an increased risk of falls [16] and disability [62].

##### Functional balance

Postural sway is a reliable [65] measure of how well a person can maintain their equilibrium during quiet standing. Increased sway indicates poor balance control and is associated with falls [64]. We will ask participants to stand as still as possible for 30 s, first with feet together and eyes open, and then with feet together and eyes closed. Lightweight, inertial wireless sensors worn on the trunk will capture area, amount, and velocity of the sway that occurs [65]. Lateral sway velocity is the outcome of interest because it may be a predictor of falls in older adults and allow for detection of changes in balance associated with cancer treatment [66, 67]. This measure will also be used to assess fidelity of the tai ji quan intervention.

##### Perceived physical function

The physical function subscale of the European Organization for Research and Treatment of Cancer Quality of Life Questionnaire (EORTC QLQ-C30) will serve as a measure of perceived physical function. This is a cancer-specific measure with scores ranging from 0 to 100, where higher scores indicate better functioning. The QLQ-C30 is commonly used in studies of prostate cancer survivors, including those done by our group [24, 68].

### Descriptive variables and additional measures of interest

*Demographic data*, *cancer history*, and *treatments* will be measured at baseline by a questionnaire created by the study team. Measures such as disease extent (e.g., presence or absence of metastatic disease) and type of ADT (e.g., intermittent vs. continuous) will be collected and updated at subsequent visits. All clinical information will be confirmed against participant electronic health records. Presence of chronic medical conditions that affect physical functioning will be measured by the Charlson Comorbidity Index and the Functional Comorbidity Index [69]. We use both instruments because Charlson provides an index of “sickness” that is commonly reported in studies of older adults, while the Functional Comorbidity Index provides a different index of the number of conditions that impact physical functioning. We will add two additional conditions to the list of 18 in the Functional Comorbidity Index to capture vestibular problems and/or peripheral neuropathy that might affect exercise ability and predispose men to fall risk in excess of ADT so that we can control for this in analyses.

*Adherence* to the exercise intervention, as measured by the percentage of prescribed sessions completed, will be tracked from attendance logs completed by the exercise instructor. Adherence data will be used to describe the dose of exercise received by participants. Make-up sessions using a written plan or video tape will not be counted in adherence estimates.

*Exercise outside the exercise intervention* could affect the fall and function outcomes in our study. In addition to using CHAMPS to measure physical activity for the frailty criterion and exercise participation during follow-up, we will also look at individual items to see whether participants significantly increase participation in other types of exercise in addition to their assigned study program.

*Fear of falling* may impact a participant’s confidence that he can safely engage in a study exercise program. This factor may also change across the intervention and is also important for describing the population. To assess fear of falling, we will use the Survey of Activities and Fear of Falling in the Elderly [70] that has 11 items representing activities of daily living associated with fear of falling, mobility, and social activities. The total score is the average of item responses, with higher scores indicating greater fear of falling.

*Perceived disability* and dependence upon others for daily tasks may result from frailty and dysfunction. If either experimental intervention reduces frailty and improves function, it may also lower the risk for disability, so that prostate cancer survivors on ADT can remain more independent. We will measure perceived disability using the 16-item disability component of the Late-Life Function and Disability Instrument (88, 89). Scores range from 0 to 100, with higher scores indicating less disability.

To capture *adverse events*, a survey will be adminstered monthly during the participants’ year-long participation in the study. If an adverse event reported through this survey indicates that their reporting condition is due to a study-related exercise activity or if more information is needed to determine reportability, study staff will follow up with a phone call or e-mail. Participants will also have the opportunity to report adverse events during exercise class or at physcial performance measurement appointments. Adverse events will be graded according to their significance for severe consequences, such as injury or death, using the following grades: mild, moderate, or severe and classified as unrelated, possibly related, or related to the study exercise programs.

### Data safety and monitoring

The OHSU Knight Cancer Institute Data and Safety Monitoring Committee (DSMC) is responsible for overall coordination of all aspects of the data safety and monitoring plan filed with the OHSU IRB. The internal audit team conducts quality assurance audits on all open clinical trials that are not monitored by another source. The initial audit will be conducted once enrollment commences and yearly thereafter. An interim safety review will occur early in the intervention period, after the first 45 enrolled men (~ 1/8th of total sample) have completed 3 months of exercise training, to assess early for program safety. The DSMC meets once each month to review the audit team’s progress and findings and to review significant adverse events and/or unanticipated problem reports, and Interim Analysis reports. The DSMC also reviews a full report of study activity for all local, active clinical trials at the time of continuing review submission including protocol amendments, revisions, consent form revisions; interim analysis results; protocol violations; total number of patients enrolled on-study as compared to expected numbers; and all unanticipated problems submitted (including dates, description, and relationship). The DSMC oversees the process of serious adverse event reporting to assure that reporting requirements are met.

### Data management and analysis plan

Standard institutional practices will be followed as described in the OHSU Information Security and Research Data Resource Guide (http://ozone.ohsu.edu/cc/sec/isg/res_sec.pdf) to maintain the confidentiality and security of data collected in this study. A copy of the consent form and documentation of consent will be maintained in the participant’s medical record as well as stored in a study file kept in a locked cabinet (for paper documents only) or stored on an encrypted and password-protected computer drive in the OHSU Knight Cancer Research Building (KCRB). All protected health information collected from the study either directly from participants or via their electronic health record will be stored on an encrypted and password-protected computer drive in the OHSU KCRB that only IRB-approved persons have access to. All other data collected for this study will be stored in OHSU installation of REDCap, a highly secure and robust web-based research data collection and management system. Any surveys that were filled out on paper will be stored in a locked cabinet in a locked, secure room in the OHSU KCRB. Data will be stored until data analysis is complete and then the data will be transferred to a repository. Access to the repository will be limited to the principal investigator, co-investigators, and associates from the original research studies. Any future human subject research study that wants to use the data from the repository will require separate IRB approval. Any future study requesting use of the data must either be related to the original research study or explore new and innovative research questions approved by the PI.

All analyses will retain each participant in the group he was randomly assigned regardless of missing data or drop out status (i.e., intention to treat; ITT). We will track medical treatment changes and disease progression during the study to describe the sample or justify why participants exited the study. In the unlikely event of a large subgroup, these data could be used for exploratory subgroup analysis to suggest directions for future research. Age, timing of ADT (current vs. past), fall history at enrollment, and baseline frailty will be considered as possible covariates in all analyses. Age and timing of ADT (past vs current) are included since they may affect exercise tolerance and/or responsiveness to exercise training; fall history is included because we want to examine the efficacy of the interventions to reduce falls in men with a fall history and prevent falls among men who have not recently fallen. Analyses will be conducted in Stata, R, and/or Mplus statistical software packages. Full information maximum likelihood estimation will be used to test the models and generate point estimates, as appropriate [71].

#### Primary outcome analysis

The efficacy of tai ji quan and strength training will be determined by comparing each of them to the control condition while adjusting for important covariates using negative binomial regression models which will analyze total number of falls per participant that occur from baseline to 6 months. A negative binomial regression was chosen over a Poisson regression for modeling the count data because of the overdispersion that is common with actual research data [21, 72]. Intervention type will be entered into the model as 2 dummy variables, with the reference group being the control group, allowing for a comparison of the each of the intervention groups against control. A second model will be run with strength training as the reference category to compare the intervention groups to each other. In additional to standard ITT analysis, the impact of participants who drop out of the study on the falls outcome will be examined in a secondary data analysis. A significant incidence rate ratio (IRR) less than 1.0 for the dummy vector representing tai ji quan and/or strength training would provide support for the hypothesis that the respective intervention reduced the rate of falls compared to the control group.

#### Secondary outcome analysis

In order to determine and compare the efficacy of tai ji quan training and strength training to reduce frailty and dysfunction, each participant will be classified as frail, pre-frail, or robust based on total frailty score. We hypothesize that the number of men classified as frail or pre-frail will decrease over the course of the study in the tai ji quan and strength training groups compared to control. Formal tests of frailty hypotheses will be conducted in a generalized mixed effects modeling framework as implemented in the R statistical computing environment [73]. Frailty category will be the multinomial dependent variable (with “robust” as the reference group), with study time point (0, 3, and 6 months), group (control as the reference group), and the group × time interactions serving as predictors [74]. We will also run a second model with strength training as the group reference category to compare the two experimental groups to each other. Significant group × time interactions will indicate that the distribution of frailty between the groups (e.g., tai ji quan vs. control) is different across time. For significant interactions, additional contrasts testing for frailty differences will be examined to describe the pattern of change across time. Group differences across time in the continuous physical function and individual frailty measures will be tested in a linear mixed effects modeling framework [73] to estimate the trajectory of change in each dependent variable across time for each individual in each group, and then comparing the average trajectories across groups. The primary effect of interest is a cross-level interaction. Specific hypotheses about treatment group effects will be specified by adding dummy variables and hypotheses will be tested through group × time interactions, as previously described here. We will also evaluate the practical significance of the interventions by summarizing results with respect to effect size.

#### Post-intervention follow-up

To determine how well the benefits of each intervention persist after structured training stops, we will use the same modeling strategies described above for each outcome (i.e., negative binomial regression, multinomial/linear mixed effects models). However, we will perform the analyses piecewise, with a change point occurring at 6 months. Of particular interest is whether the observed effects for the treatment groups differ or remain the same after the change point. In addition, we will examine whether participation in exercise following the end of the intervention moderates the persistent effect of the intervention on study endpoints. We will categorize the mode of exercise participants engaged in (i.e., walking/aerobic, strength training, tai ji quan, stretching) for at least 50% of the follow-up period. These categories will then be dummy coded and entered into the analyses outlined above, along with the product of these dummy vectors and treatment to represent the interaction. A significant coefficient for the product term would indicate that continued exercise modifies the effect of the intervention on falls, frailty, or physical function. This analysis will allow us to determine whether continued participation in an exercise program maintained improvements for an intervention group, or a lack of participation caused a loss of improvements in an intervention group, and whether control participants who begin programs similar to the intervention programs improve in outcomes. In addition, linear and logistic regression models will be used to explore different predictors (e.g., age, comorbidities, timing of ADT) of adherence to exercise after structured training stops.

#### Analysis to determine responders to interventions

We will use a growth mixture modeling (GMM) approach using Mplus to explore if there are distinct types of men (based on age, baseline frailty, cancer stage, ADT duration and timing, other treatments) who benefit most from the intervention(s). GMM is a type of clustering technique that can simultaneously handle longitudinal data and multiple measures to identify distinct subgroups of patients who have different responses to an intervention. For longitudinal outcomes, we will employ GMM to identify distinct patterns of change in subpopulations with varying response (growth) trajectories and unique variances reflecting homogenous within-trajectory growth. Patients will be assigned to the “most likely class” or pattern of change over time (e.g., men who improve most from an intervention), and follow-up analyses (e.g., multinomial regression) will be conducted to describe the demographic and clinical characteristics of patients who are classified into different subgroups.

### Dissemination

The dissemination of this trial will occur through the registration of the trial with ClinicalTrials.gov within 21 days of enrollment of the first study participant and posting of primary trial results on ClinicalTrials.gov within 1 year of study completion.

## Discussion

Prostate cancer survivors treated with ADT are at increased risk for frailty and falls, and there are no evidence-based treatments to combat these risks. Tai ji quan and strength training interventions have both been shown to reduce fall rates in older adults, but these exercise modalities have never been tested in a large randomized, controlled trial in prostate cancer survivors treated with ADT. Since it is unknown if balance or strength deficits may contribute more to fall risk in this population, it is important to test exercise modalities that impact these deficits against each other, and against an active control (i.e., flexibility) exercise group. In addition, balance or strength training may reduce the rate of aging processes that are likely accelerated by ADT use, which also contribute to fall risk.

Our primary hypotheses are based on preliminary data indicating that falls and frailty are significantly elevated by exposure to ADT and that therapeutically tailored fall prevention interventions have the potential for beneficial effects on the important outcomes of our investigation. Our head-to-head comparison of two exercise countermeasures to the newly uncovered threats of falls and frailty to PC survivors’ is methodologically robust, cost and time effective, and the first of its kind to be conducted in any group of male cancer survivors**.** To our knowledge, this will be the first study to evaluate the impact of two distinct exercise interventions in PC survivors with either falls or frailty as intended clinical endpoints. Including a follow-on period and identifying characteristics of responders to the interventions is also novel because we will be the first to evaluate outcomes that provide information about how to implement fall prevention programs in oncology practice. Each study intervention is based upon training programs for the general population of older adults created by members of our team and which have been implemented in community settings. Thus, our interventions could be readily translated into clinical practice using similar implementation methods.

The potential impact of this study could be large because nearly half of all prostate cancer survivors may be treated with ADT [2] and median survival time following diagnosis for these older men is 16 years [75–77]. Falls, frailty, and dysfunction lead to costly injuries, loss of independence, and early death among older persons. Exercise represents the only potential non-pharmacologic countermeasure to these life-threatening side effects of ADT. In order to advance exercise as a prescriptive therapy to fall and frailty prevention the efficacy of each modality must first be established in this rigorous controlled trial. Our study addresses an unmet need by testing potentially low-cost, safe, scalable exercise-based solutions that could ultimately prevent or delay the falls and frailty linked to ADT. Identifying the type of exercise that best reduces frailty and dysfunction from ADT may also reduce disability risk, thereby prolonging independence and survival [78]. In 2011, the first generation of baby boomers turned 65 and the aging of this generation contributes to the projected doubling of cancer survivors by 2050 [79]. Healthy People 2020 set a goal to reduce illness, disability, and death caused by cancer [80] and the proposed study can make a significant contribution toward this goal.

## Trial status

At the date of publication, the current protocol version is 10.0. Recruitment for the trial began in January 2019 and is expected to complete in January 2023.

## Supplementary Information

**Additional file 1:** IRB approved protocol.

**Additional file 2:** NIH notice of grant award.

**Additional file 3:** Timeline of events for participants in the trial.

## Data Availability

The datasets used during the current study are available from the corresponding author on reasonable request.

## Abbreviations

ADT: Androgen deprivation therapy
ACSM: American College of Sports Medicine
BIA: Bioelectrical impedance analysis
CHAMPS: Community Healthy Activities Model Program for Seniors
DSMC: Data and Safety Monitoring Committee
DXA: Dual-energy X-ray absorptiometry
EORTC QLQ-C30: European Organization for Research and Treatment of Cancer Quality of Life Questionnaire
FACIT: Functional Assessment of Chronic Illness Therapy
GMM: Growth mixture modeling
ITT: Intent to treat
KCRB: Knight Cancer Research Building
MET: Metabolic equivalent
OHSU: Oregon Health & Science University
OSCaR: Oregon State Cancer Registry
PC: Prostate cancer
REDCap: Research Electronic Data Capture
TUG: Timed up and go
