# Supplementary material for: Protocol for GET FIT Prostate: a randomized, controlled trial of group exercise training for fall prevention and functional improvements during and after treatment for prostate cancer
Source: Trials. 2021 Nov 6;22:775. doi: 10.1186/s13063-021-05687-7 (PMC8571824; doi:10.1186/s13063-021-05687-7)
Supplement: Supplementary file 1 — Additional file 1: IRB approved protocol. [file 13063_2021_5687_MOESM1_ESM.pdf]

OREGON HEALTH & SCIENCE UNIVERSITY

IRB MEMO

Research Integrity Office
3181 SW Sam Jackson Park Road - L106RI
Portland, OR 97239-3098
(503)494-7887 irb@ohsu.edu

APPROVAL OF SUBMISSION

May 8, 2018

Dear Investigator:

On 4-24-2018, the IRB reviewed the following submission:

IRB ID:	STUDY00018354
Type of Review:	Initial Study
Title of Study:	Group Exercise Training for Fall prevention and functional Improvements during and after Treatment for Prostate cancer
Principal Investigator:	Kerri Winters
Funding:	Name: DHHS NIH Natl Cancer Inst, PPQ #: 1013066
IND, IDE, or HDE:	None
Documents Reviewed:	• Consent and Authorization Form<br>• CoIR - management plan for IRB study 18354 . 4.6.2018<br>• Recruitment - Research Match Notice.pdf<br>• Knight CRRC Submission Coverletter_signed.pdf<br>• Prostate Questionnaire MSQ<br>• GETFITProstate6fullNIHgrant.pdf<br>• GET FIT Prostate Use-of-Ionizing-Radiation-in-Humans-Form.docx<br>• HIPAA-Prep-to-Research-Form-FINAL-3-5-2014.docx<br>• Prostate Questionnaire - Post Intervention Survey<br>• HIPAA - WOA.doc<br>• Prostate PPQ KWS VT signed<br>• CRRC Administrative Review - Approved<br>• Protocol<br>• Knight DSMP v5.1_2017.03.14.pdf<br>• Questionnaire - GET FIT Prostate Phone Screen.pdf<br>• Prostate Questionnaire 9&12 month Follow-up exercise habits<br>• Biostatistics Approval Form<br>• Prostate Physician Clearance<br>• IRB 18354_initial submission response

	memo_20180426.docx<br>• Prostate Questionnaire - Main Survey Packet

The IRB granted final approval on 5/8/2018. The study is approved until 4/23/2019.

Review Category: Full Board

Copies of all approved documents are available in the study's **Final** Documents (far right column under the documents tab) list in the eIRB. Any additional documents that require an IRB signature (e.g. IIAs and IAAs) will be posted when signed. If this applies to your study, you will receive a notification when these additional signed documents are available.

Ongoing IRB submission requirements:

- Six to ten weeks before the expiration date, you are to submit a continuing review to request continuing approval.
- Any changes to the project must be submitted for IRB approval prior to implementation.
- Reportable New Information must be submitted per OHSU policy.
- You must submit a continuing review to close the study when your research is completed.

Guidelines for Study Conduct

In conducting this study, you are required to follow the guidelines in the document entitled, "Roles and Responsibilities in the Conduct of Research and Administration of Sponsored Projects," as well as all other applicable OHSU IRB Policies and Procedures.

Requirements under HIPAA

If your study involves the collection, use, or disclosure of Protected Health Information (PHI), you must comply with all applicable requirements under HIPAA. See the HIPAA and Research website and the Information Privacy and Security website for more information.

IRB Compliance

The OHSU IRB (FWA00000161; IRB00000471) complies with 45 CFR Part 46, 21 CFR Parts 50 and 56, and other federal and Oregon laws and regulations, as applicable, as well as ICH-GCP codes 3.1-3.4, which outline Responsibilities, Composition, Functions, and Operations, Procedures, and Records of the IRB.

Sincerely,

The OHSU IRB Office
