# Supplementary material for: Protocol for GET FIT Prostate: a randomized, controlled trial of group exercise training for fall prevention and functional improvements during and after treatment for prostate cancer
Source: Trials. 2021 Nov 6;22:775. doi: 10.1186/s13063-021-05687-7 (PMC8571824; doi:10.1186/s13063-021-05687-7)
Supplement: Supplementary file 2 — Additional file 2: NIH notice of grant award. [file 13063_2021_5687_MOESM2_ESM.pdf]

RESEARCH
Department of Health and Human Services
National Institutes of Health

NATIONAL CANCER INSTITUTE

Federal Award Date: 06/05/2020

Grant Number: 5R01CA222605-03
FAIN: R01CA222605

Principal Investigator(s):
KERRI M WINTERS-STONE, PHD

Project Title: Preventing Falls and Frailty in Prostate Cancer Survivors: GET FIT Prostate

Derby, Mark
Grants & Contracts Admin - S
3181 SW Sam Jackson Park Rd
L106OPAM
Portland, OR 972393098

Award e-mailed to: orserv@ohsu.edu

Period Of Performance:
Budget Period: 07/01/2020 – 06/30/2021
Project Period: 07/23/2018 – 06/30/2023

Dear Business Official:

The National Institutes of Health hereby awards a grant in the amount of $643,607 (see “Award Calculation” in Section I and “Terms and Conditions” in Section III) to OREGON HEALTH & SCIENCE UNIVERSITY in support of the above referenced project. This award is pursuant to the authority of 42 USC 241 42 CFR 52 and is subject to the requirements of this statute and regulation and of other referenced, incorporated or attached terms and conditions.

Acceptance of this award including the “Terms and Conditions” is acknowledged by the grantee when funds are drawn down or otherwise obtained from the grant payment system.

Each publication, press release, or other document about research supported by an NIH award must include an acknowledgment of NIH award support and a disclaimer such as “Research reported in this publication was supported by the National Cancer Institute of the National Institutes of Health under Award Number R01CA222605. The content is solely the responsibility of the authors and does not necessarily represent the official views of the National Institutes of Health.” Prior to issuing a press release concerning the outcome of this research, please notify the NIH awarding IC in advance to allow for coordination.

Award recipients must promote objectivity in research by establishing standards that provide a reasonable expectation that the design, conduct and reporting of research funded under NIH awards will be free from bias resulting from an Investigator’s Financial Conflict of Interest (FCOI), in accordance with the 2011 revised regulation at 42 CFR Part 50 Subpart F. The Institution shall submit all FCOI reports to the NIH through the eRA Commons FCOI Module. The regulation does not apply to Phase I Small Business Innovative Research (SBIR) and Small Business Technology Transfer (STTR) awards. Consult the NIH website http://grants.nih.gov/grants/policy/coi/ for a link to the regulation and additional important information.

If you have any questions about this award, please contact the individual(s) referenced in Section IV.

Sincerely yours,

Tracie McGraw
Grants Management Officer
NATIONAL CANCER INSTITUTE

Additional information follows

SECTION I – AWARD DATA – 5R01CA222605-03

Award Calculation (U.S. Dollars)

**Federal Direct Costs**	$449,432
**Federal F&A Costs**	$194,175
**Approved Budget**	$643,607
**Total Amount of Federal Funds Obligated (Federal Share)**	$643,607
**TOTAL FEDERAL AWARD AMOUNT**	$643,607
**AMOUNT OF THIS ACTION (FEDERAL SHARE)**	$643,607

SUMMARY TOTALS FOR ALL YEARS		
**YR**	**THIS AWARD**	**CUMULATIVE TOTALS**
3	$643,607	$643,607
4	$637,906	$637,906
5	$645,859	$645,859

Recommended future year total cost support, subject to the availability of funds and satisfactory progress of the project

Fiscal Information:
CFDA Name: Cancer Detection and Diagnosis Research
CFDA Number: 93.394
EIN: 1931176109A1
Document Number: RCA222605A
PMS Account Type: P (Subaccount)
Fiscal Year: 2020

IC	CAN	2020	2021	2022
CA	8479566	$643,607	$637,906	$645,859

Recommended future year total cost support, subject to the availability of funds and satisfactory progress of the project

NIH Administrative Data:
PCC: H6HP / **OC**: 41025 / **Released**: MCGRAWT 06/04/2020
Award Processed: 06/05/2020 12:37:18 AM

SECTION II – PAYMENT/HOTLINE INFORMATION – 5R01CA222605-03

For payment and HHS Office of Inspector General Hotline information, see the NIH Home Page at http://grants.nih.gov/grants/policy/awardconditions.htm

SECTION III – TERMS AND CONDITIONS – 5R01CA222605-03

This award is based on the application submitted to, and as approved by, NIH on the above-titled project and is subject to the terms and conditions incorporated either directly or by reference in the following:

a. The grant program legislation and program regulation cited in this Notice of Award.
b. Conditions on activities and expenditure of funds in other statutory requirements, such as those included in appropriations acts.
c. 45 CFR Part 75.
d. National Policy Requirements and all other requirements described in the NIH Grants Policy Statement, including addenda in effect as of the beginning date of the budget period.
e. Federal Award Performance Goals: As required by the periodic report in the RPPR or in the final progress report when applicable.
f. This award notice, INCLUDING THE TERMS AND CONDITIONS CITED BELOW.

(See NIH Home Page at http://grants.nih.gov/grants/policy/awardconditions.htm for certain references cited above.)

Research and Development (R&D): All awards issued by the National Institutes of Health (NIH) meet the definition of "Research and Development" at 45 CFR Part§ 75.2**.** As such, auditees should identify NIH awards as part of the R&D cluster on the Schedule of Expenditures of Federal Awards (SEFA). The auditor should test NIH awards for compliance as instructed in Part V, Clusters of Programs. NIH recognizes that some awards may have another classification for purposes of indirect costs. The auditor is not required to report the disconnect (i.e., the award is classified as R&D for Federal Audit Requirement purposes but non-research for indirect cost rate purposes), unless the auditee is charging indirect costs at a rate other than the rate(s) specified in the award document(s).

This institution is a signatory to the Federal Demonstration Partnership (FDP) Phase VI Agreement which requires active institutional participation in new or ongoing FDP demonstrations and pilots.

An unobligated balance may be carried over into the next budget period without Grants Management Officer prior approval.

This grant is subject to Streamlined Noncompeting Award Procedures (SNAP).

This award is subject to the requirements of 2 CFR Part 25 for institutions to receive a Dun & Bradstreet Universal Numbering System (DUNS) number and maintain an active registration in the System for Award Management (SAM). Should a consortium/subaward be issued under this award, a DUNS requirement must be included. See http://grants.nih.gov/grants/policy/awardconditions.htm for the full NIH award term implementing this requirement and other additional information.

This award has been assigned the Federal Award Identification Number (FAIN) R01CA222605. Recipients must document the assigned FAIN on each consortium/subaward issued under this award.

Based on the project period start date of this project, this award is likely subject to the Transparency Act subaward and executive compensation reporting requirement of 2 CFR Part 170. There are conditions that may exclude this award; see http://grants.nih.gov/grants/policy/awardconditions.htm for additional award applicability information.

In accordance with P.L. 110-161, compliance with the NIH Public Access Policy is now mandatory. For more information, see NOT-OD-08-033 and the Public Access website: http://publicaccess.nih.gov/.

This award provides support for one or more clinical trials. By law (Title VIII, Section 801 of Public Law 110-85), the "responsible party" must register "applicable clinical trials" on the ClinicalTrials.gov Protocol Registration System Information Website. NIH encourages registration of all trials whether required under the law or not. For more information, see http://grants.nih.gov/ClinicalTrials_fdaaa/

In accordance with the regulatory requirements provided at 45 CFR 75.113 and Appendix XII to 45 CFR Part 75, recipients that have currently active Federal grants, cooperative agreements, and procurement contracts with cumulative total value greater than $10,000,000 must report and maintain information in the System for Award Management (SAM) about civil, criminal, and administrative proceedings in connection with the award or performance of a Federal award that reached final disposition within the most recent five-year period. The recipient must also make semiannual disclosures regarding such proceedings. Proceedings information will be made publicly available in the designated integrity and performance system (currently the Federal Awardee Performance and Integrity Information System (FAPIIS)). Full reporting requirements and procedures are found in Appendix XII to 45 CFR Part 75. This term does not apply to NIH fellowships.

Treatment of Program Income:

Additional Costs

Clinical Trial Indicator: Yes
This award supports one or more NIH-defined Clinical Trials. See the NIH Grants Policy Statement Section 1.2 for NIH definition of Clinical Trial.

REQUIREMENT: The clinical trial(s) supported by this award is subject to the plan submitted to NIH and the NIH policy on Dissemination of NIH-Funded Clinical Trial Information. The plan states that the clinical trial(s) funded by this award will be registered in ClinicalTrials.gov not later than 21 calendar days after enrollment of the first participant and primary summary results reported in ClinicalTrials.gov, not later than one year after the completion date. The reporting of summary results is required by this term of award even if the primary completion date occurs after the period of performance.

REQUIREMENT: This award is subject to additional certification requirements with each submission of the Annual, Interim, and Final Research Performance Progress Report (RPPR). The recipient must agree to the following annual certification when submitting each RPPR. By submitting the RPPR, the AOR signifies compliance, as follows:

In submitting this RPPR, the SO (or PD/PI with delegated authority), certifies to the best of his/her knowledge that, for all clinical trials funded under this NIH award, the recipient and all investigators conducting NIH-funded clinical trials are in compliance with the recipient's plan addressing compliance with the NIH Policy on Dissemination of NIH-Funded Clinical Trial Information. Any clinical trial funded in whole or in part under this award has been registered in ClinicalTrials.gov or will be registered not later than 21 calendar days after enrollment of the first participant. Summary results have been submitted to ClinicalTrials.gov or will be submitted not later than one year after the completion date, even if the completion date occurs after the period of performance.

REQUIREMENT: The awardee is required to follow the data and safety monitoring plan included in the application and may not implement any changes in the plan without the written prior approval of the National Cancer Institute.

INFORMATION: This award involves Human Subjects Research. See "Assurance Requirements and Institutional Review Boards" under Part II, Subpart A, Human Subjects, in the NIH Grants Policy Statement, for specific requirements and grantee responsibilities related to the protection of human subjects, which are applicable to and are a term and condition of this award.

This award reflects the National Cancer Institute's acceptance of the certification that all key personnel have completed education on the protection of human subjects, in accordance the NIH Grants Policy Statement, "Education in the Protection of Human Research Subjects."

Any individual involved in the design and conduct of the study that is not included in the certification must satisfy this requirement prior to participating in the project. Failure to comply can result in the suspension and/or termination of this award, withholding of support of the continuation award, audit disallowances, and/or other appropriate action.

STAFF CONTACTS

The Grants Management Specialist is responsible for the negotiation, award and administration of this project and for interpretation of Grants Administration policies and provisions. The Program Official is responsible for the scientific, programmatic and technical aspects of this project. These individuals work together in overall project administration. Prior approval requests (signed by an Authorized Organizational Representative) should be submitted in writing to the Grants Management Specialist. Requests may be made via e-mail.

Grants Management Specialist: Tracie Mcgraw
Email: mcgrawth@mail.nih.gov **Phone**: (301) 631-3020 **Fax**: 301-451-5391

Program Official: Frank Perna
Email: pernafm@mail.nih.gov **Phone**: (240) 276-6782

SPREADSHEET SUMMARY
GRANT NUMBER: 5R01CA222605-03

INSTITUTION: OREGON HEALTH & SCIENCE UNIVERSITY

Facilities and Administrative Costs	Year 3	Year 4	Year 5
F&A Cost Rate 1	54%	54%	54%
F&A Cost Base 1	$359,584	$355,882	$363,732
F&A Costs 1	$194,175	$192,176	$196,415
