# Supplementary material for: Protocol for GET FIT Prostate: a randomized, controlled trial of group exercise training for fall prevention and functional improvements during and after treatment for prostate cancer
Source: Trials. 2021 Nov 6;22:775. doi: 10.1186/s13063-021-05687-7 (PMC8571824; doi:10.1186/s13063-021-05687-7)
Supplement: Supplementary file 3 — Additional file 3: Timeline of events for participants in the trial. [file 13063_2021_5687_MOESM3_ESM.docx]

Participant Timeline

| **Months in Study** | **0** | **3** | **6** | **12** |
| --- | --- | --- | --- | --- |
| Enrollment | x |  |  |  |
| Intervention | x | x | x |  |
| Assessments | x | x | x | x |
